# Supplementary material for: Unsymmetrical Trifluoromethyl Methoxyphenyl β-Diketones: Effect of the Position of Methoxy Group and Coordination at Cu(II) on Biological Activity
Source: Molecules. 2021 Oct 26;26(21):6466. doi: 10.3390/molecules26216466 (PMC8588221; doi:10.3390/molecules26216466)
Supplement: Supplementary file 1 [file molecules-26-06466-s001.zip › molecules-1387532-supplementary.pdf]

Unsymmetrical trifluoromethyl methoxyphenyl β-diketones: effect of the position of methoxy group and coordination at Cu(II) on the biological activity

Liliya A. Khamidullina [1,2], Igor S. Puzyrev *,[1], Gennady L. Burygin [3], Pavel V. Dorovatovskii [4], Yan V. Zubavichus [5], Anna V. Mitrofanova [6], Victor N. Khrustalev [6,7], Tatiana V. Timofeeva [8], Pavel A. Slepukhin [1], Polina D. Tobysheva [2], Alexander V. Pestov [1,2], Euro Solari[9], Alexander G. Tskhovrebov *,[6], Valentine G. Nenajdenko *,[10]

Table S1. Crystallographic parameters, data collection and structure refinement details for H**L1** and **1**–**3**.

Compound	H**L1**	**1**	**2**	**3**
Chemical formula	$C_{11}H_9F_3O_3$	$C_{25}H_{23}CuF_6NO_7$	$C_{22}H_{16}CuF_6O_6$	$C_{24}H_{22}CuF_6O_7S$
Formula weight	246.18	626.98	553.90	632.03
Temperature (K)	100(2)	295(2)	100(2)	100(2)
Crystal system	monoclinic	monoclinic	triclinic	monoclinic
Space group	$P2_1/c$	$P2_1/c$	P-1	$P2_1/c$
a (Å)	9.642(3)	13.6154(5)	6.3548(13)	13.455(3)
b (Å)	11.088(4)	12.3213(8)	9.5537(19)	11.866(2)
c (Å)	9.951(3)	16.4123(11)	19.231(4)	15.874(3)
α (°)	90.00	90.00	84.06(3)	90.00
β (°)	98.042(4)	91.841(4)	84.77(3)	91.53(3)
γ (°)	90.00	90.00	73.31(3)	90.00
V (Å^3)	1053.4(6)	2751.9(3)	1110.0(4)	2533.5(9)
Z	4	4	2	4
D_{calc} (g cm^{-3})	1.552	1.513	1.657	1.657
μ (mm^{-1})	0.146	0.878	2.420	1.432
F(000)	504	1276	558	1284
Θ range (°)	2.133– 32.323	3.8490–29.4280	1.445–38.336	3.420–30.937
Index ranges	$-13 \leq h \leq 13$	$-18 \leq h \leq 17$	$-7 \leq h \leq 7$	$-17 \leq h \leq 17$
	$-16 \leq k \leq 16$	$-16 \leq k \leq 9$	$-12 \leq k \leq 11$	$-15 \leq k \leq 15$
	$-14 \leq l \leq 14$	$-21 \leq l \leq 21$	$-24 \leq l \leq 24$	$-20 \leq l \leq 17$
Measured reflections	16815	19757	17686	32650
Independent reflections	3576	7340	4719	5532
Observed reflections [I ≥ 2σ(I)]	3074	4647	3593	5136
Final R indices [I > 2σ(I)]	R_1 = 0.0425, wR_2 = 0.1221	R_1 = 0.0580, wR_2 = 0.1748	R_1 = 0.0490, wR_2 = 0.1117	R_1 = 0.0364, wR_2 = 0.0968
R indices [all data]	R_1 = 0.0482, wR_2 = 0.1275	R_1 = 0.1024, wR_2 = 0.2421	R_1 = 0.0705, wR_2 = 0.1270	R_1 = 0.0389, wR_2 = 0.0988
GOF (F^2)	1.074	1.029	0.982	1.065
Max., min. Δρ (e Å^{-3})	0.501, –0.519	1.082, –0.525	0.671, –0.899	0.430, –0.778

Table S2. Selected interatomic distances (Å) and angles (°) of **1**–**3**.

Interatomic distance	**1**	**2**	**3**
Cu1—O1	1.926(2)	1.9358(17)	1.9496(14)
Cu1—O1^{i}		1.9358(17)	
Cu1—O2	1.929(3)	1.9504(16)	1.9331(14)
Cu1—O2^{i}		1.9505(16)	
Cu1—O3	1.933(2)		
Cu1—O4	1.937(2)		1.9286(14)
Cu1—O5	2.339(3)		1.9363(14)
Cu1—O7			2.2946(15)
Angle			
O1—Cu1—O1^{i}		180.0	
O1—Cu1—O2	93.05(10)	93.18(7)	92.50(6)
O1^{i} —Cu1—O2^{i}		93.18(7)	
O1—Cu1—O2^{i}		86.82(7)	
O1^{i} —Cu1—O2		86.82(7)	
O2—Cu1—O2^{i}		180.0	
O1—Cu1—O3	85.77(10)		
O2—Cu1—O3	172.58(13)		
O1—Cu1—O4	174.08(11)		87.25(6)
O2—Cu1—O4	88.13(10)		172.09(6)
O3—Cu1—O4	92.31(10)		
O1—Cu1—O5	95.11(11)		170.67(6)
O2—Cu1—O5	93.93(13)		86.30(6)
O3—Cu1—O5	93.47(13)		
O4—Cu1—O5	90.58(11)		92.67(6)
O4—Cu1—O7			93.45(6)
O2—Cu1—O7			94.46(6)
O5—Cu1—O7			95.96(6)
O1—Cu1—O7			93.35(6)

Symmetry codes: (i) $-x+2$, $-y+1$, $-z$.

Table S3. Bond lengths (Å) and angles (°) in the hydrogen-bonding scheme for H**L1**.

Donor –H··· Acceptor	D–H	H···A	D···A	D–H···A
O(1)–H(1)···O(2)	0.927(18)	1.627(18)	2.4956(14)	154.3(16)
O(1)–H(1)···F(3)^{i}	0.927(18)	2.467(17)	2.8555(15)	105.3(12)
C(2)–H(2)···F(3)	0.95	2.41	2.7361(17)	100
C(6)–H(6)···O(1)	0.95	2.38	2.7112(15)	100
C(11)–H(11A)···O(1)^{ii}	0.98	2.60	3.4272(18)	142

Symmetry code: (i) 2–x,1/2+y,3/2–z, (ii) 1–x,–1/2+y,1/2–z

Table S4. Bond lengths (Å) and angles (°) in the hydrogen-bonding scheme for **1**.

Donor –H··· Acceptor	D–H	H···A	D···A	D–H···A
C(3)–H(3A)···F(3)	0.93	2.36	2.728(5)	103
C(10)–H(10A)···O(1)	0.93	2.38	2.711(5)	101
C(14)–H(14A)···F(4)	0.93	2.36	2.734(7)	104
C(17)–H(17A)···O(3)	0.93	2.40	2.720(5)	100
C(23)–H(23A)···O(4)	0.93	2.53	3.105(6)	120
C(24)–H(24A)···O(5)	0.96	2.33	2.741(9)	105

Table S5. Bond lengths (Å) and angles (°) in the hydrogen-bonding scheme for **3**.

Donor –H··· Acceptor	D–H	H···A	D···A	D–H···A
C(2)–H(2)···F(1)	0.95	2.37	2.743(2)	103
C(9)–H(9)···O(5)^{i}	0.95	2.52	3.225(3)	131
C(13)–H(13)···F(4)	0.95	2.34	2.723(2)	103
C(21)–H(21)···O(5)	0.95	2.39	2.720(2)	100

Symmetry code: (i) *1–x,–1/2+y,3/2–z*

Table S6. Bond lengths (Å) and angles (°) in the hydrogen-bonding scheme for **2**.

Donor –H··· Acceptor	D–H	H···A	D···A	D–H···A
C(3)–H(3)···F(1)	0.95	2.38	2.755(3)	103
C(6)–H(6)···O(2)	0.95	2.43	2.754(3)	100
C(14)–H(14)···F(5)	0.95	2.39	2.762(3)	103
C(17)–H(17)···O(5)	0.95	2.43	2.756(3)	100
C(20)–H(20)···O(6)^{i}	0.95	2.57	3.494(3)	165

Symmetry code: (i) *–1–x,1–y,1–z*

Table S7. Maximal inhibition values at corresponding concentrations of test compounds towards the cancer (HeLa) and normal (Vero) cell lines that determined in this work.

Test compounds	Maximal inhibition, % (at concentration, µM)	
	Vero	HeLa
H**L1**	35 (406 µM)	42 (536 µM)
H**L0**	46 (406 µM)	33 (536 µM)
cis-[Cu(**L1**)$_2$(DMSO)]	46 (158 µM)	62 (791 µM)
cis-[Cu(**L0**)$_2$(DMSO)$_2$]	47 (141 µM)	98 (2112 µM)

Table S8. Minimum inhibitory concentration (μg·mL^{-1}/mM) for reference compounds (adapted from [1–3]).

Test organism	H**L0** [1]	*cis*-[Cu(**L0**)$_2$(DMSO)$_2$] [1]	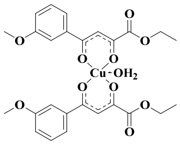 [2]	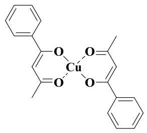 [3]
S. aureus ATCC 25923	256/1.04	128/0.20	781/1.35	13/0.03
S. aureus ATCC 29213	512/2.08	128/0.20	–	–
B. subtilis ATCC 6633	64/0.26	64/0.10	781/1.35	14/0.04
E. coli ATCC 25922	512/2.08	256/0.41	1562/2.69	13/0.03
P. atrosepticum RCAM 01724	128/0.52	128/0.20	–	–
P. atrosepticum 34-1/1	128/0.52	64/0.10	–	–
C. albicans	256/1.04	512/0.81	195/0.34	–

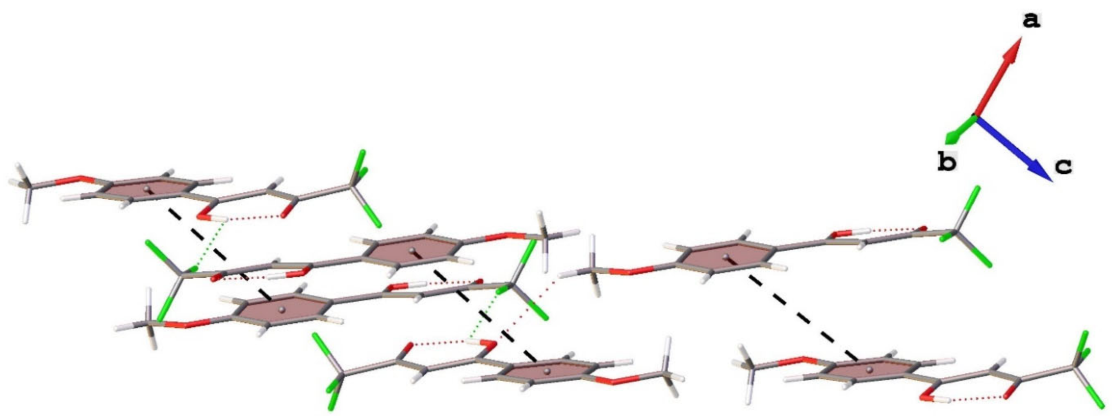

Figure S1. The molecular packing fragment of the H**L1**.

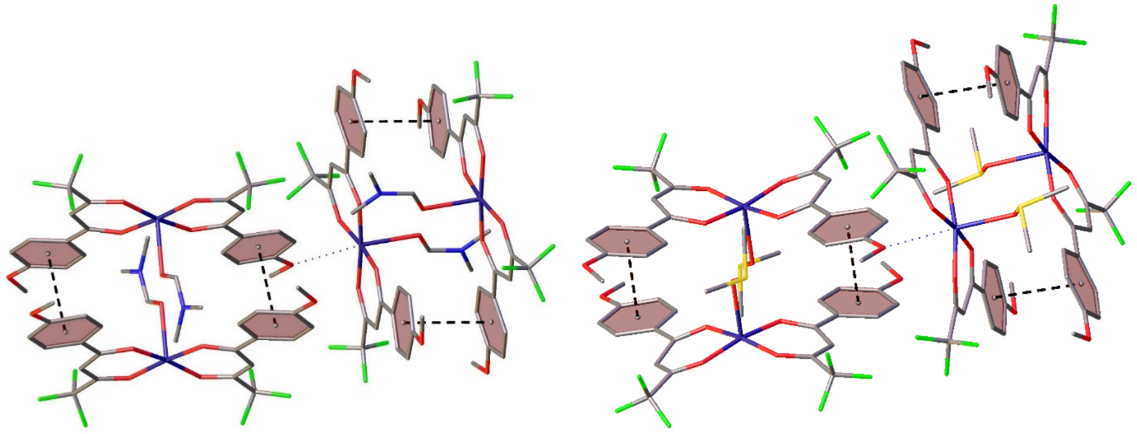

Figure S2. Partial packing diagram of the **1** (left) and **3** (right), showing molecules linked into dimers. For the sake of clarity, H atoms and minor position of disordered groups are omitted.

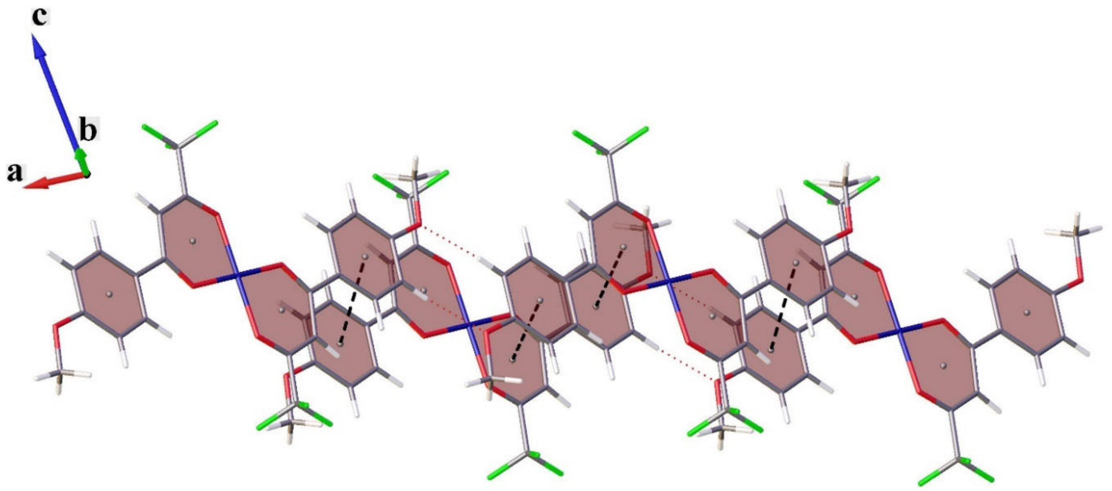

Figure S3. The molecular packing fragment of the **2**.

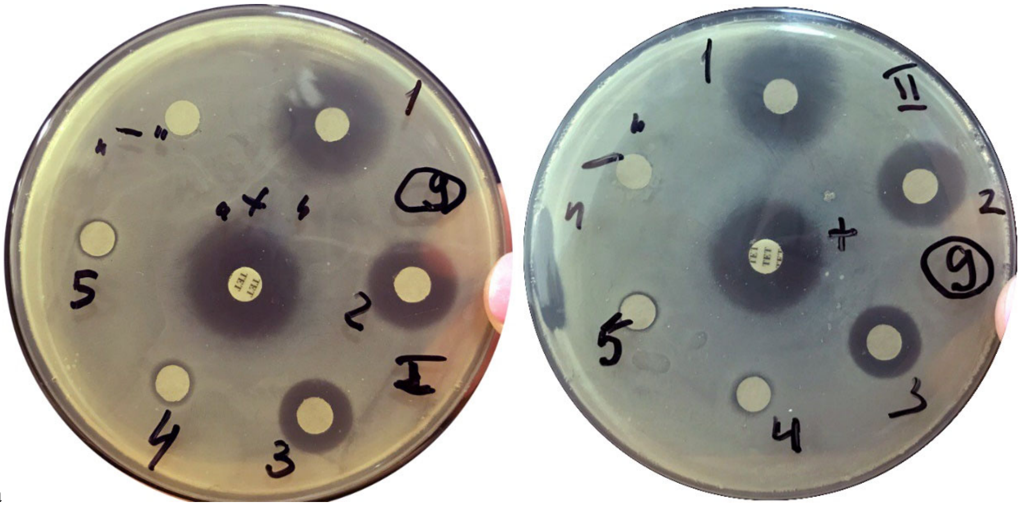

a

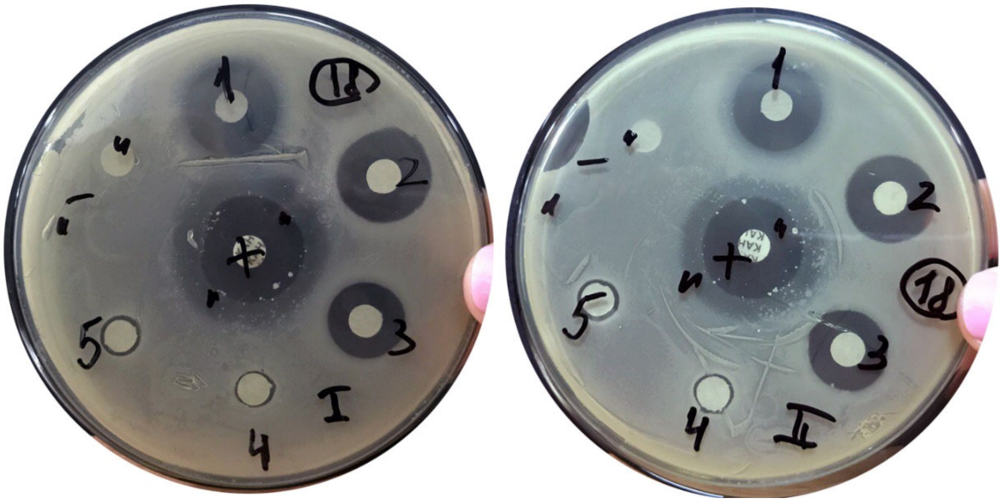

b

Figure S4. Paper disks containing test compound H**L1** (labeled as 1) shown on an agar plate of bacteria (two replicates are labeled as I and II). Circular zones of bacterial growth inhibition surrounded some disks, indicating susceptibility to the compounds. Tetracycline and kanamycin (labeled as "+") served as positive controls for *S. aureus* ATCC 25923 (a) and *B. subtilis* ATCC 6633 (b), respectively. Solvent (labeled as "–") served as negative control.

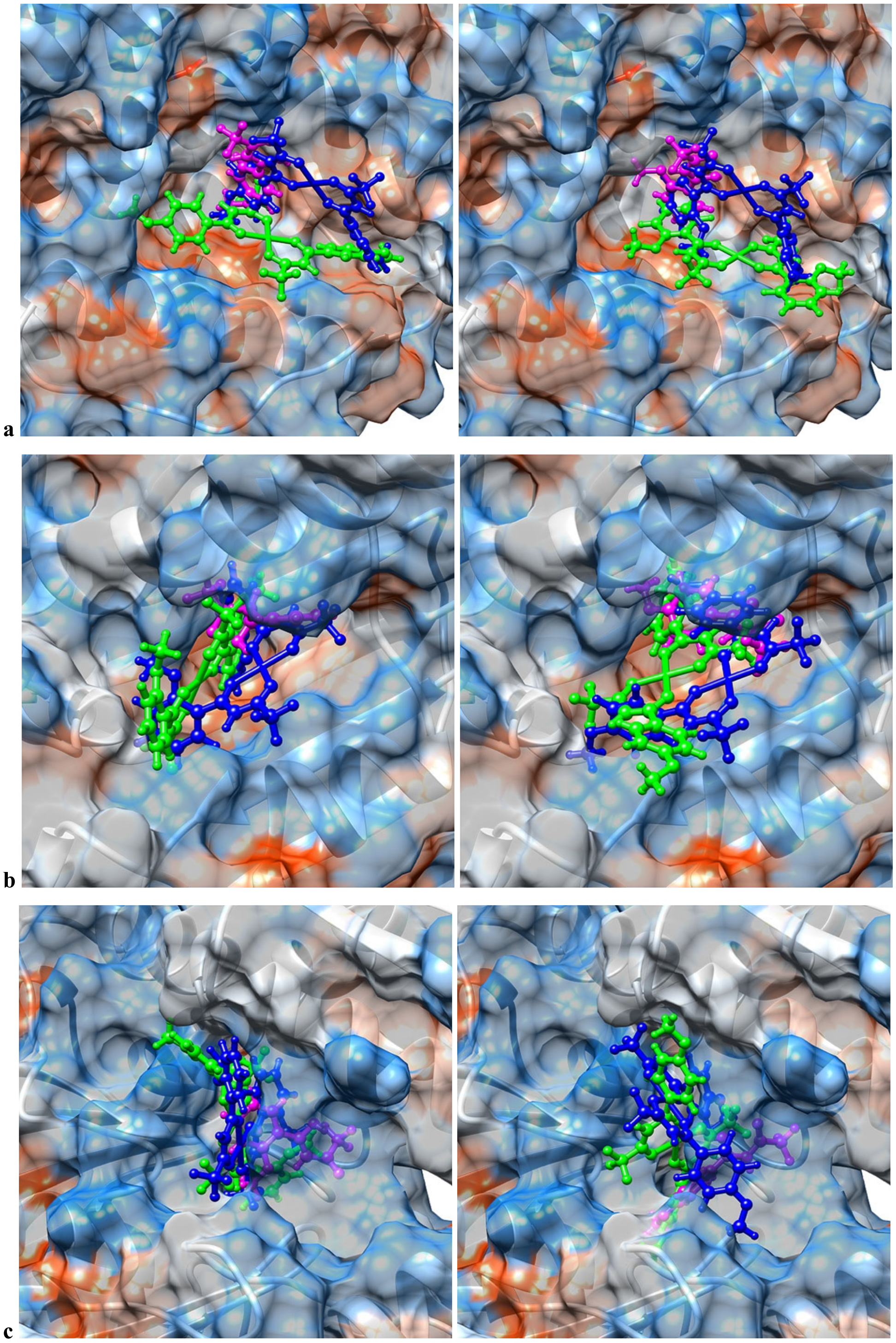

Figure S5. Comparison of the binding positions of the ligands and their metal complexes (H**L0**, *cis*-[Cu(**L0**)$_2$], *trans*-[Cu(**L0**)$_2$] – left, H**L1**, *cis*-[Cu(**L1**)$_2$], *trans*-[Cu(**L1**)$_2$] – right) perched on the surface of the active sites of RNR (a), Hsp90 (b), Hsp70 (c). The molecular surfaces of binding pockets are displayed, as well as the conformation of ligands H**L0**, H**L1** (magenta stick), *trans*-[Cu(**L0**)$_2$], *trans*-[Cu(**L1**)$_2$] (green stick) and *cis*-[Cu(**L0**)$_2$], *cis*-[Cu(**L1**)$_2$] (cyan stick) in the binding pocket. The coloring of the surfaces is from hydrophilic (blue) to hydrophobic (red).

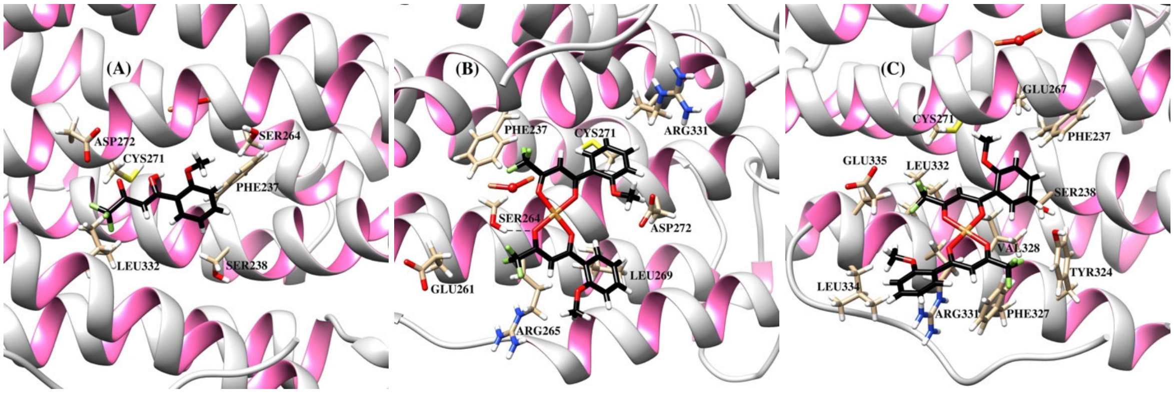

Figure S6. Ribbon representation of the R2 RNR protein with bound compounds H**L0** (a), *cis*-[Cu(**L0**)$_2$] (b), *trans*-[Cu(**L0**)$_2$] (c). Binding site showing selected amino acids and hydrogen bonding (dashed line) between the compound *cis*-[Cu(**L0**)$_2$] and Ser264.

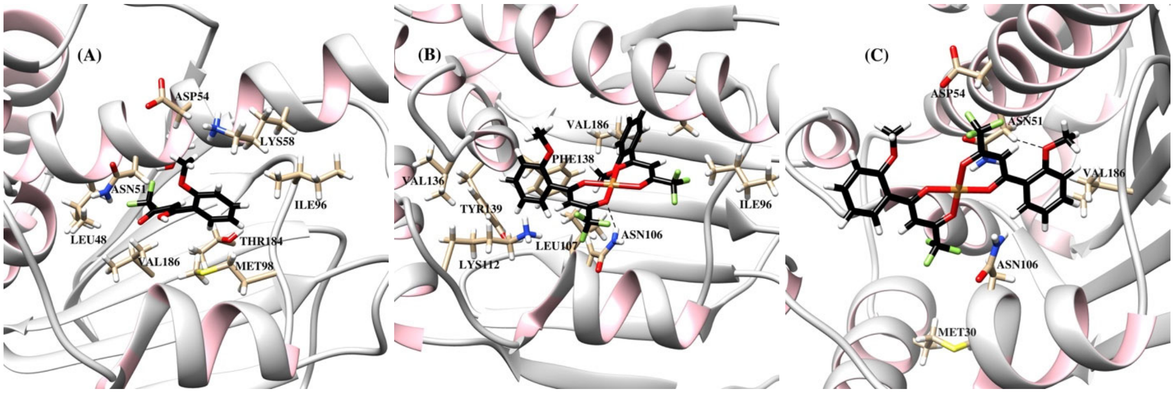

Figure S7. Ribbon representation of the Hsp90 protein with bound compounds H**L0** (a), *cis*-[Cu(**L0**)$_2$] (b), *trans*-[Cu(**L0**)$_2$] (c). Binding site showing selected amino acids and hydrogen bonding (dashed lines) between the compounds and Asn106, Asn51.

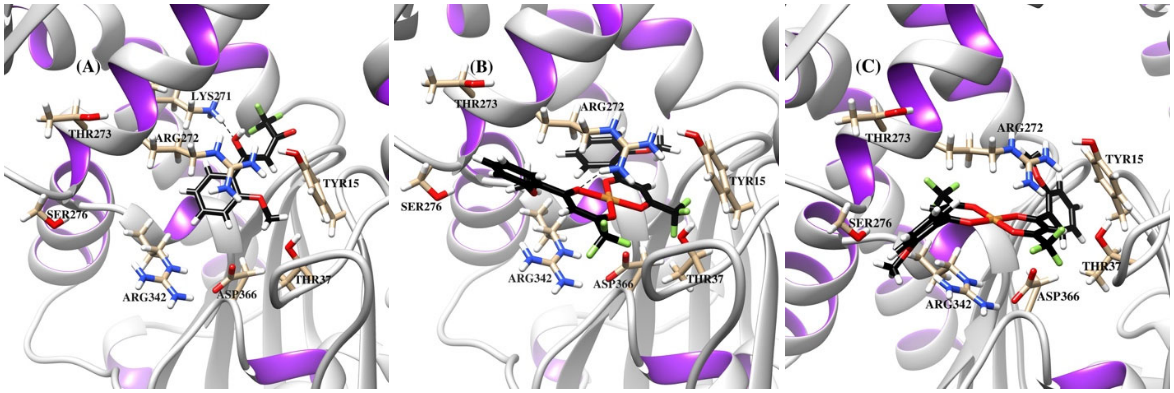

Figure S8. Ribbon representation of the Hsp70 protein with bound compounds H**L0** (a), *cis*-[Cu(**L0**)$_2$] (b), *trans*-[Cu(**L0**)$_2$] (c). Binding site showing selected amino acids and hydrogen bonding (dashed lines) between the compounds and Lys271, Arg272.

References

1. L.A. Khamidullina, I.S. Puzyrev, T.V. Glukhareva, S.A. Shatunova, P.A. Slepukhin, P.V. Dorovatovskii, Y.V. Zubavichus, V.N. Khrustalev, Z. Fan, T.A. Kalinina, A.V. Pestov, J. Mol. Struct. 1176 (2019) 515–528. doi: 10.1016/j.molstruc.2018.08.112.

2. N. Joksimović, D. Baskić, S. Popović, M. Zarić, M. Kosanić, B. Ranković, T. Stanojković, S.B. Novaković, G. Davidović, Z. Bugarčić, N. Janković, Dalt. Trans. 45 (2016) 15067–15077. doi: 10.1039/C6DT02257J.

3. H.M. Krishnegowda, C.S. Karthik, M.H. Marichannegowda, K. Kumara, P.J. Kudigana, M. Lingappa, P. Mallu, L.K. Neratur, Inorganica Chim. Acta, 484 (2019) 227–236. doi: 10.1016/j.ica.2018.09.049.
